# A comparison of antenatal classifications of ‘overweight’ and ‘obesity’ prevalence between white British, Indian, Pakistani and Bangladeshi pregnant women in England; analysis of retrospective data

**DOI:** 10.1186/s12889-017-4211-1

**Published:** 2017-04-11

**Authors:** Rebecca Garcia, Nasreen Ali, Andy Guppy, Malcolm Griffiths, Gurch Randhawa

**Affiliations:** 1 Institute for Health Research, The University of Bedfordshire, Putteridge Bury, Hitchin Road, Luton, UK; 2 The Institute for Applied Social Sciences, The University of Bedfordshire, Park Square, Luton, Bedfordshire UK; 3 The Luton & Dunstable University Hospital NHS Foundation Trust, Lewsey Rd, Luton, Bedford, LU4 0DZ UK

**Keywords:** South Asian, Pakistani, Indian Bangladeshi, BMI, Maternal obesity, White British

## Abstract

**Background:**

Maternal obesity increases women’s risk of poor birth outcomes, and statistics show that Pakistani and Bangladeshi women (who are born or settled) in the UK experience higher rates of perinatal mortality and congenital anomalies than white British or white Other women. This study compares the prevalence of maternal obesity in Indian, Pakistani, Bangladeshi and white British women using standard and Asian-specific BMI metrics.

**Method:**

Retrospective cross-sectional analysis using routinely recorded secondary data in Ciconia Maternity information System (CMiS), between 2008 and 2013. Mothers (*n* = 15,205) whose ethnicity was recorded as white British, Bangladeshi, Pakistani or Indian. Adjusted standardised residuals and Pearson Chi-square. Main outcome measures: Percentage of mothers stratified by ethnicity (Indian, Pakistani, Bangladeshi and white British) who are classified as overweight or obese using standard and revised World Health Organisation BMI thresholds.

**Results:**

Compared to standard BMI thresholds, using the revised BMI threshold resulted in a higher prevalence of obesity: 22.8% of Indian and 24.3% of Bangladeshi and 32.3% of Pakistani women. Pearson Chi-square confirmed that significantly more Pakistani women were classified as ‘obese’ compared with white British, Indian or Bangladeshi women (*χ* ^*2*^ = 499,88 *df* = 9, *p* < 0.001).

**Conclusions:**

There are differences in the prevalence of obese and overweight women stratified by maternal ethnicity of white British, Indian, Pakistani and Bangladeshi. Using revised anthropometric measures in Indian, Pakistani and Bangladeshi women has clinical implications for identifying risks associated with obesity and increased complications in pregnancy.

## Background

Existing evidence shows that South Asian women in the United Kingdom (UK) (i.e. Indian, Pakistani, Bangladeshi women) [1] have higher rates of adverse birth outcomes, including perinatal mortality, compared with white British or white Other mothers [2, 3]. Studies have shown that obesity is associated with increased risks of maternal comorbidity [4–6] congenital anomaly [7] and stillbirth [8]. Therefore, maternal obesity increases a mother’s risk profile during pregnancy. Moreover, the health Survey for England (HSE) demonstrated that more Asian women (i.e. Indian, Pakistani, Bangladeshi and Chinese), when compared with white women, were identified at ‘increased risk’ of health complications when reporting BMI using revised thresholds for Asian individuals [9]. However, much of the existing research on maternal obesity utilises the World Health Organisation (WHO) standard thresholds of BMI, despite revised thresholds being published by WHO, for use in South Asians [10–12].

The World Health Organisation (WHO) categorises a BMI equal or greater than 25 as ‘overweight’, equal or greater than 30 as ‘obese’ , and equal than or less than 18.5 as ‘underweight’. Scholars have long debated the accuracy of a standard BMI being applicable across all ethnicities. In 2004, WHO determined that the existing BMI thresholds were inappropriate to be applied to individuals of Asian ethnicity, as the increased risk of adverse morbidity has been found to occur in lower BMI classifications [13, 14]. Subsequently, guidance has been published that identifies lower risk thresholds; namely: 23–27.5 kg/m^2^ as ‘*increasing risk*’ (or overweight) and greater than 27.7 kg/m^2^ as ‘*increased*’ risk (or obese) [11, 15]. These are aimed to identify health risks earlier, such as diabetes, cardiovascular disease and hyperlipidaemia, which are known to occur in higher prevalence in individuals of South Asian origin [10, 11]. At the time of writing, the revised guidance on BMI for Asian women has not been incorporated into the NICE guidance for antenatal care or NICE guidance on diabetes in pregnancy, suggesting that pregnant South Asian women are currently less likely to be identified at higher risk of pregnancy complications, though a raised BMI.

The contribution of maternal obesity in adverse birth outcomes is only beginning to become known, with a recent study using routine collected data in London (2004–2012) demonstrated that obese women of South Asian origin (i.e. Indian, Pakistani, Bangladeshi, Asian Other) were 2.4 times more likely to have a stillbirth than white British women [3]. To our knowledge, only one previous published paper addresses the issue of BMI metrics in pregnant South Asian mothers [16]. The authors found that on the application of revised BMI thresholds, the prevalence of obese South Asian women increased from 18.8% to 30.9%. However, the South Asian women included within this study were Pakistani; therefore it is unclear whether Indian and Bangladeshi pregnant mothers would show the same BMI trend.

The recently published maternity review has emphasised the need for improved maternal care for all sections of the UK’s diverse population [17]. Consequently, enabling accurate identification of at-risk populations is essential for personalised care and targeted interventions as recommended [17, 18]. This paper contributes to the paucity of current studies that implement the revised BMI thresholds for South Asian women in an antenatal setting. It aims to compare the prevalence of maternal overweight and obesity in women of South Asian origin (i.e. Indian, Pakistani and Bangladeshi) in Luton, England, using revised standard and BMI thresholds, to determine whether there are any differences.

## Methods

### Design and study population

Retrospective*,* cross-sectional analysis of routinely collected *secondary data from Ciconia Maternity information System* (CMiS) at the Luton and Dunstable University Hospital was used. CMiS is a clinical information system, used in some NHS maternity units. Purposive sampling of women aged 16 and over, who delivered their infants between January 2008 and December 2013 was conducted, extracting a number of pre-identified variables. Specific postcode areas were targeted, in order to ensure that the purposive sample was geographically homogenous, since the hospital accepts maternity referrals from a large geographical area across the East of England. The data extract pertained to all deliveries that met the inclusion criteria and for the purpose of this study; the cases were stratified by ethnicity. Ethic approval was given by the University of Bedfordshire Research Ethics Committee (March 2014). The hospitals Information Governance Manager ensured adherence to patient confidentiality and data protection before de-identified routinely collected data was provided.

### Variables

Following a review of the existing literature, pre-determined variables were extracted from CMiS as part of a larger study. The variables of maternal height (m), weight (kg), BMI (kg/m^2^), ethnicity (i.e. White British, Indian, Pakistani and Bangladeshi) were used in this analysis. Recording the patient/clients ethnicity in the NHS is mandatory, which is achieved by asking the individual their self-ascribed ethnic category (which incorporates ancestry, shared language, culture, and religion) [19] and is aligned to 2001 census categories [25]. Therefore, maternal country of birth or length of residence was not established for this study. Maternal height, weight and BMI are recorded in CMiS following the first antenatal consultation (known as the ‘booking visit), and typically occurs before 12 weeks of gestation.

### Statistical analysis

Analyses were conducted using IBM Statistics Package for the Social Sciences (SPSS)® v21. The raw data contained data on all ethnicities (*N* = 21,264). White British, Pakistani, Indian and Bangladeshi outcomes were extracted and are reported in this paper (*n* = 15,203).

The variables were initially inspected using frequency counts by each ethnic group and 3 BMI outliers were subsequently removed, to avoid distorting the data. There were *n* = 8 missing BMI cases. The BMI variable was re-coded into two new catagorical variables; according to standard BMI thresholds (<18 underweight, 18–24 healthy weight, 25–29 overweight, 30–39 obese, 40+ extremely obese) and the revised WHO BMI (2004) thresholds for individuals of South Asian origin; (18–23 increasing risk, 23–27.5 increased risk, 27.5+ high risk). The output for height was provided in metric and imperial measurements; therefore a manual conversion was required, to transform all imperial measurements into metric. This was achieved using syntax code in SPSS. There were *n* = 8 cases of missing data for BMI, *n* = 2475 missing cases of height. Weight was reported in kilograms, with *n* = 2201 missing cases. Missing cases were deleted.

Means and standard deviations were calculated for each group, and cross-tabulation was used with maternal ethnicity and BMI (standard and revised). Since there were unequal sample sizes between the groups, and the assumptions of linearity and homogeneity of variance were violated, Pearson Chi-square and Adjusted Standardised Residuals (ASR) were selected as the most appropriate tests to determine if there was any significant independence between ethnicity and standard or revised BMI classifications [20, 21, 22]. ASR uses a standardised score and helps to identify where in the contingency table of ethnicity x BMI threshold (standard and revised) the data deviated (from expected counts) and was either under or over represented (i.e. +/− 0.96, *p* = <0.05).

## Results

There was a total of 15,203 recorded BMI’s in CMiS for 2008–2013, recorded from White British, Indian, Pakistani and Bangladeshi women. The database cohort mean BMI was 25.81 kg/m^2^. Descriptive statistics of this cohort’s weight, height and BMI at booking is detailed in Table 1. This shows that Bangladeshi mothers have the lightest mean weight (60.26 kg) compared to white British mothers who have the heaviest mean weight (70.33 kg). The cohort mean weight is 66.88 kg, showing that white British expectant mothers have above mean weights, while Indian, Pakistani and Bangladeshi mothers have below mean weights.

**Table 1:** Descriptive statistics of maternal weight by cohort and mothers ethnicity

Group		N	Minimum	Maximum	Mean	Std. deviation
Whole cohort	Weight	13,010	30.45	191.00	66.88	15.57
	Height	12,736	1.00	165.50	153.99	43.16
	BMI Booking	15,208	.00	99.00	21.90	11.23
	Valid N (listwise)	12,420				
White British	Weight	6094	30.45	191.00	70.33	16.70
	Height	6011	1.00	187.00	155.10	48.33
	BMI Booking	7019	.00	77.00	21.84	11.12
	Valid N (listwise)	5875				
Indian	Weight	873	35.00	108.00	61.73	11.93
	Height	862	1.51	181.00	154.51	26.59
	BMI Booking	991	.00	45.00	20.67	9.84
	Valid N (listwise)	840				
Pakistani	Weight	4307	32.95	141.00	65.72	14.19
	Height	4127	1.50	165.50	153.55	41.31
	BMI Booking	5141	.00	52.00	20.21	11.70
	Valid N (listwise)	4034				
Bangladeshi	Weight	1736	35.00	166.00	60.26	12.84
	Height	1736	1.52	165.50	150.89	34.03
	BMI Booking	2057	.00	99.00	20.26	10.85
	Valid N (listwise)	1671				

Descriptive statistics for maternal BMI (at booking) by ethnicity is also shown in Table 1. This demonstrates that white British women have the highest mean BMI (26.21 kg/m^2^), while Indian mothers have the lowest (24.47 kg/m^2^). Tables 2 and 3 depict maternal BMI by maternal ethnicity using standard and revised classifications. This shows that using standard classifications, the highest percent of ‘overweight’ (i.e. BMI 25 kg/m^2^) is seen in white British mothers (43%) compared to the lowest percentage of Indian mothers (36.2%). However, when the data is reclassified, using the revised WHO thresholds, 49% of Pakistani mothers are identified ‘at increased risk’ (obese); while the percentage of Indian mothers remains lower at 45.7%, slightly higher than white British percentages for ‘overweight’ (43% using standard measures).﻿ Table 4 shows the percentage of women classified as 'obese' or 'high risk', according to the BMI thresholds applied. ﻿

**Table 2:** Maternal ethnicity and BMI frequencies using standard classifications

BMI standard n (%)							
Maternal ethnicity	N	<18	18–25	25–30	30–39	40+	Total over 25(%)
White British	7015	1367(19.50)	2540(36.20)	1747(24.90)	1173(16.7)	188(2.70)	3038(43)
Indian	991	205(20.70)	427(44.30)	269(27.10)	82(8.30)	8(0.80)	359(36.20)
Pakistani	5141	1296(25.20)	1644(32)	1313(25.50)	807(15.70)	81(1.60)	2201(42.8)
Bangladeshi	2056	488(23.70)	762(37.10)	553(26.90)	228(11.10)	25(1.20)	806(39.20)
Total (N)	15,203						6474(42.50)

**Table 3:** Maternal ethnicity and BMI frequencies using revised WHO classifications

BMI *revised for South Asian* n (%)						
Maternal ethnicity	n	<18.5	18.5–23	23–27.5	27.5	Total over 23(%)
Indian	991	205(20.70)	333(33.60)	264(26.60)	189(19.10)	453(45.7)
Pakistani	5141	1296(25.20)	1322(25.70)	1226(23.80)	1297(25.20)	2523(49)
Bangladeshi	2056	488(23.7)	616(30)	550(26.80)	402(19.60)	952(46.3)
Total (N)	8188					3928(47.9)

**Table 4:** Maternal ethnicity and prevalence of obesity

		BMI standard n (%)	BMI for South Asian n (%)
Maternal ethnicity	n	Total over 30	Total over 27.5
White British	5549	1186(21.30%)	^a^
Indian	816	86(10.54%)	187(22.80%)
Pakistani	3977	878(22.07%)	1289(32.30%)
Bangladeshi	1652	252(15.25%)	402(24.30)

^a^Application of revised BMI is only intended for individuals of ‘Asian’ ethnicity

A Pearson Chi-Square test was conducted to determine independence between ethnicity and the revised BMI categories. The result was statistically significant (χ2 = 499.88 *df* = 9, *p* < 0.001) and suggests strongly that BMI (coded into these bands) is significantly associated with maternal ethnicity. Adjusted Standardized Residuals (ASR) were estimated for each cell in the Chi-square analysis to reveal significant areas of over and under-representation. The results showed that white British women were significantly over-represented in the 18-25 kg/m^2^ group (ASR = 20.2), in contrast to Pakistani mothers who were significantly under-represented in the 18.5-23 kg/m^2^ group (ASR = −15.8). Furthermore, the results showed that both Indian (ASR = −1.8) and Bangladeshi (ASR = −1.2) mothers were slightly (not significantly) underrepresented in the >27.5 kg/m^2^ group, whereas, Pakistani mothers were significantly over-represented (ASR = 12.1). Similarly, while all South Asian mothers were over-represented in the <18.5 kg/m^2^ group, Indian (ASR = 3.6) and Bangladeshi (ASR = 5.4) were considerably more represented than Pakistani mothers (ASR = 2.4).

## Discussion

Using data that was routinely collectedover six years, this paper reports the differences found in the prevalence of maternal BMI between White British, Indian, Pakistani and Bangladeshi women in Luton, when applying the revised BMI thresholds compared to standard BMI thresholds. The results show that white British women have the heaviest mean weight (kg), and when applying standard BMI metrics, they have the highest mean BMI (kg/m^2^); while Bangladeshi women have the lightest mean weight and Indian women have the lowest mean BMI. Moreover, when using standard BMI thresholds, a higher percentage of white British mothers are found to be classified as ‘overweight’ (>25 kg/m^2^), followed by Pakistani women, while Indian women are found to be the least overweight having the smallest percentage of mothers classified as overweight within this cohort. However, when applying the revised WHO BMI thresholds (white British women excluded) the prevalence percentage of women identified at ‘increasing risk’ is higher for Indian, Pakistani and Bangladeshi women. Consequently, when using the revised BMI metrics for South Asian women, a higher prevalence of mothers are identified at-risk when compared to using standard BMI metrics, and the numbers at risk are higher than the prevalence of obese white British mothers. In other words, more South Asian women are at risk from pregnancy adversity as a consequence of being overweight or obese than the prevalence of white British mothers, when using the correct BMI metrics per maternal ethnicity.

Differences between the maternal ethnic groups were checked, and statistical significance was confirmed, showing that Indian and Bangladeshi mothers are more likely (compared to chance) to be found in the lower BMI categories (<18.5 kg/m^2^), while Pakistani mothers were found to be underrepresented in the lower BMI range and overly represented in BMI >27.5 kg/m^2^.

Comparing the revised BMI thresholds in this study has found that more mothers of Indian, Pakistani and Bangladeshi ethnicity are identified as ‘*increased risk;* of health consequences related to cardiovascular disease and diabetes than using the current standard BMI measure [11].

In addition, the outcomes from this study confirm that comparisons of BMI between mothers of South Asian ethnicity are indeed heterogenic. Current UK clinical guidelines (i.e. National Institute for health and Care Excellence) use the standard WHO BMI thresholds. Therefore a small but important number of South Asian women, who fall between 27 and 30 kg/m^2^ will not be identified as being at higher risk. Remarkably, for Indian women within this sample, the prevalence of identified at-risk women are over double using <27 kg/m^2^, than when using >30 kg/m^2^ as an identifier.

The results from this study are similar to those reported by Bryant et al. (2014), insofar that this cohort showed a similar prevalence of a raised BMI (32.3%) as that found in the Born in Bradford study, which demonstrated a prevalence rate of 30.9% in Pakistani women [16]. The reasons for this are unclear. Furthermore, a few studies have highlighted that a greater prevalence of South Asian pregnant women is found to be underweight [23–25]. Indeed, this study also supports that Indian and Bangladeshi mothers are more likely to be underweight, compared with Pakistani mothers, which further demonstrates important differences between Indian, Pakistani and Bangladeshi mothers, supporting the opinion that data incorporating South Asian people should not be aggregated together, since the risks and management of being underweight or overweight are different.

Accurate identification of maternal risk factors in pregnancy will help reduce adverse birth outcomes. Research has shown that maternal underweight also leads to increased risk of complications including low birthweight and preterm birth [4, 26]. Moreover, the prevalence of having a low birthweight infant is a high in South Asian mothers in the UK. However, the precise mechanisms remain unclear. These results show Indian and Bangladeshi mothers may be underweight; a low body mass contributes towards increased pregnancy risks of a low birthweight infant or delivery preterm.

Similarly, maternal overweight and obesity are also known to mediate adverse birth outcomes; including stillbirth [3]. Research has shown that there is an increased risk of stillbirth [3] and congenital anomalies [7] in obese mothers. Furthermore, Penn and colleagues (2014) found that South Asian ethnicity and obesity was an independent risk factor for stillbirth. Statistics have shown an increased incidence of stillbirth in Pakistani mothers compared to other ethnic groups [27]. In clinical terms, early and accurate identification of all risk factors that are detrimental to maternal and foetal health should be considered, and appropriate intervention and management applied. Therefore, as found in this study, the highest prevalence of overweight and at-risk mothers was seen the Pakistani mothers, it could be asserted that a higher number of Pakistani mothers are at risk through a raised BMI than is currently identified using standard thresholds. Interestingly, within the raw data, maternal height and weight at booking were the two variables representing the most missing data on all the collected variables. Although the reason for the missing data is not known, compared to the missing data frequency of ethnicity, (which is part of the NHS mandatory data set), it is clear that between the first antenatal appointment, recording of height and weight and transference of data into CMiS, some anthropometric data is incomplete.

This study is the first to our knowledge to apply and compare the revised BMI thresholds for South Asian individuals, in the UK with standard BMI thresholds, bringing attention to important differences between pregnant South Asian mothers (i.e. Indian, Pakistani and Bangladeshi) and similar risk factors for maternal overweight and underweight. The current study acknowledges an important limitation. It was not possible to extract maternal age in this dataset and therefore maternal age has not been controlled for. It is known that maternal weight and risk of adversity increases with maternal age [8, 28]. Future research should consider how the identified differences between South Asian mothers might impact on adverse birth outcomes, which will help us understand the underlying mechanisms more clearly.

## Conclusion

This study compared the standard BMI and revised BMI thresholds in an antenatal setting. The results found a higher percentage of South Asian women were classified at increased risk (obese) than when using standard BMI thresholds (BMI > 25 kg/m^2^). This has potential implications for both policy and practice.

## Abbreviations

ASR: Adjusted standard residuals
CMiS: Ciconia Maternity information System
HSE: Health Survey for England
NICE: National Institute for Health and Care Excellence
WHO: World Health Organisation
